# Immunotherapeutic potential of varicella vaccine in smoldering and cutaneous adult T-cell leukemia/lymphoma

**DOI:** 10.1186/1742-4690-11-S1-P21

**Published:** 2014-01-07

**Authors:** M Amano, M Setoyama

**Affiliations:** 1 Department of Dermatology, University of Miyazaki, Kiyotake, Miyazaki, Japan

## 

There are several subtypes of HTLV-1-induced adult T-cell leukemia/lymphoma (ATLL): acute, lymphoma, chronic, and smoldering. Chronic and smoldering ATLL have a relatively good prognosis, even without treatment. However, these types can evolve to acute type ATLL, which has a poor prognosis. Previously we experienced some ATLL patients with improving skin involvements after suffering from herpes zoster and we reported that varicella-zoster virus could be capable of activating the immune system by the manifestation of active inflammation. We postulated that varicella vaccine could be a novel immunotherapeutic agent against ATLL. According to the original protocol, patients received a biweekly administration of varicella vaccine. In this retrospective study, 33 patients were recruited in our institute between March 2002 and December 2010. All patients with a previously untreated and histology proven ATLL were eligible for the purpose of the study. Complete response lasting for six months or more was seen in 6 (18%), partial response in 2 (6%), and no response in 7 (21%) patients, with a median follow-up duration of 27 months (range, 6-99 months). The median overall survival was 24 months and this was significantly longer compared to previously reported *f*indings (16 months). No serious adverse events were observed. In conclusion, smoldering ATLL with the skin lesions and cutaneous ATLL are promising candidates for this varicella vaccine immunotherapy.

